# Digital Skills and Readiness of Greek Nurses for Artificial Intelligence Adoption in Clinical Nursing Practice

**DOI:** 10.3390/nursrep16040129

**Published:** 2026-04-11

**Authors:** Nikolaos Kontodimopoulos, Ioanna Anagnostaki, Kejsi Ramollari, Alexandra Anna Gasparinatou, Michael A. Talias

**Affiliations:** 1 Department of Economics and Sustainable Development, Harokopio University, 176 76 Athens, Greece; 2 School of Social Sciences, Hellenic Open University, 263 31 Patras, Greece; joanna10xs@yahoo.gr (I.A.); keisyramollari@gmail.com (K.R.); 3 Nursing Service, General University Hospital of Patras, 265 04 Patras, Greece; 4 Department of Informatics and Telematics, Harokopio University, 177 78 Athens, Greece; alegas@hua.gr; 5 Healthcare Management Program, School of Economics & Management, Open University of Cyprus, 2220 Nicosia, Cyprus; michael.talias@ouc.ac.cy

**Keywords:** artificial intelligence, nursing education, digital skills, readiness for AI, technology adoption, digital health, nursing workforce

## Abstract

**Background:** Artificial intelligence (AI) is increasingly integrated into healthcare systems, with important implications for nursing practice and clinical workflows. However, evidence regarding nurses’ digital skills, perceptions, and readiness to adopt AI-enabled technologies remains limited, particularly in national healthcare contexts such as Greece. **Objectives:** This study examined nurses’ digital skills, perceptions of AI, and readiness for AI adoption in clinical practice, and explored demographic and professional factors associated with these outcomes. **Methods:** A cross-sectional survey was conducted among 166 nurses working in two public hospitals in Greece. **Results:** Nurses reported moderate digital skills, with 59.1% indicating competence in email/video communication and 27.2% reporting adequate use of digital security tools, while exposure to AI remained limited (18.0% reported using AI products/services in daily life). Perceived professional impact of AI was moderate, whereas readiness for AI adoption was comparatively lower, with only 7.8% considering health professionals adequately prepared and 7.2% reporting adequate AI training. Statistical analyses indicated that educational level and computer literacy certification were positively associated with digital skills, whereas longer professional experience was negatively associated with readiness for AI adoption. **Conclusions:** These findings highlight a gap between general digital competence and preparedness for AI-driven healthcare applications and underline the need for targeted education and implementation strategies to support effective and ethical integration of AI in nursing practice. From a nursing workforce perspective, the results underscore the importance of integrating AI literacy into continuing professional education and aligning digital health implementation strategies with clinical nursing practice.

## 1. Introduction

Artificial Intelligence (AI) is among the most transformative technological developments of recent decades and is increasingly shaping scientific research, industry, and society. One of the earliest definitions describes AI as a scientific field that combines engineering and computer science for the development of intelligent applications [1]. More broadly, AI refers to the ability of algorithms embedded in systems and tools to learn from data, enabling them to perform automated tasks without the explicit step-by-step programming traditionally required by humans [2]. In this sense, AI encompasses systems capable of mimicking core human cognitive functions, such as learning, reasoning, problem solving, and decision-making [3].

AI is expected to have a significant impact on healthcare, particularly in clinical decision support, diagnostics, care delivery, and healthcare system management [2,3,4]. These applications highlight AI’s potential to improve clinical outcomes while enhancing efficiency, safety, and sustainability within healthcare systems. In the context of the present study, AI is considered at a conceptual and implementation level, focusing on clinical adoption and workforce readiness rather than on the technical development or evaluation of specific machine learning algorithms.

One of the most established applications of AI in healthcare is automated diagnosis in medical imaging, where AI systems have contributed to improved diagnostic accuracy, efficiency, and decision-making [5,6,7]. In magnetic resonance imaging, AI models demonstrate the ability to predict pathological features and therapeutic response [8], while in cardiovascular imaging, AI-based approaches have been shown to enhance the diagnosis of cardiovascular diseases [9,10]. In addition, AI supports a range of digital health applications, including electronic health (e-Health), mobile health (m-Health), and assistive technologies for individuals with diverse healthcare needs [11]. AI-based approaches are also increasingly applied to physiological and biosignal data (e.g., ECG, EEG, and vital signs) for clinical monitoring and decision support [12].

Beyond clinical applications, AI technologies are used in areas such as surgical robotics and pharmaceutical development, contributing to improved precision, process optimization, and innovation in healthcare delivery [13,14]. Despite these advances, important challenges remain, particularly regarding transparency, reliability, and accountability in clinical use [2,5,6].

### 1.1. Artificial Intelligence in Nursing Care: Evidence, Readiness and Research Gap

The integration of AI into the nursing profession introduces new opportunities and advances in care delivery and plays a central role in supporting nursing decision-making [15,16,17]. Nurses can take timely and informed actions through AI-supported predictive analytics, monitoring (e.g., intravenous transfusions, epileptic seizures, heart rate, critical alarms, and mortality), and the prediction of infections and pressure ulcers [15,16,18]. Such monitoring applications are increasingly linked with nurse-assisted remote patient monitoring interventions, enabling continuous surveillance and timely clinical response in everyday nursing practice [19]. Remote monitoring interventions further enable effective communication and collaboration between patients and nurses, promote person-centered care, transform nursing workflows, and strengthen patient self-management. Patient self-management encompasses care based on both nurse and patient experience, as well as technological nursing competence [19].

Beyond general attitudes toward AI, the adoption of AI-enabled technologies in nursing practice depends strongly on the digital competence of the workforce [20,21,22]. Digital competence in nursing refers not only to technical operational skills but also to information literacy, clinical data interpretation, responsible digital communication, and awareness of ethical and legal requirements in technology-supported care [23,24]. International frameworks in nursing informatics emphasize that these competencies are essential prerequisites for the effective use of digital health tools and for the safe integration of AI in clinical workflows [18,25,26]. Therefore, assessing nurses’ digital skills is increasingly viewed as a necessary step for identifying workforce readiness and designing targeted training interventions [27,28].

Empirical studies and recent reviews indicate that nurses often perceive AI as potentially beneficial for decision support, monitoring, and workflow optimization [29,30,31]; however, readiness for adoption remains uneven and is influenced by multiple barriers and facilitators [16,18,32]. Frequently reported barriers include limited AI education and training opportunities, lack of familiarity with AI concepts, concerns regarding accountability and patient safety, low trust in automated decision-making, and fear that AI may alter professional roles [28,33,34]. Facilitators include perceived usefulness, previous exposure to digital health technologies, organizational support, leadership endorsement, and the availability of clear governance and ethical guidance [28,35,36]. These findings highlight that AI implementation in nursing requires both technological readiness and human-centered strategies that strengthen competence and trust [37,38]. Within nursing science, AI readiness is increasingly conceptualized as part of broader professional digital competence and clinical decision-support preparedness.

Despite increasing interest in AI-supported clinical practice, a significant gap remains in the evidence regarding nurses’ digital skills and readiness to adopt AI technologies, particularly within the national healthcare system of Greece [3,39,40,41]. In addition, few studies have jointly examined AI readiness alongside sociodemographic and professional factors, training exposure, and self-reported digital competence [20,29,42]. This study addresses this gap by assessing the digital skills and readiness of Greek nurses for the adoption of AI in clinical practice.

Against this background, the present study provides context-specific empirical evidence on Greek nurses’ digital skills and readiness for AI adoption, while also exploring the demographic and professional factors associated with readiness, thereby addressing an important gap in the literature.

### 1.2. Digital Competence, Technology Acceptance, and Study Objectives

Digital technology skills are defined as a set of knowledge, abilities, and attitudes that enhance users’ digital competence by facilitating access to information and data, the creation and dissemination of digital content, effective communication and collaboration within digital environments, as well as security management and problem solving [43,44]. Regarding healthcare, such skills are essential for the safe and effective adoption of AI-driven technologies. Consequently, digital competence is increasingly viewed as a core professional capability within contemporary nursing education and lifelong learning frameworks.

Nurses’ perceptions regarding the acceptance and use of AI are particularly important, given the wide range of AI-based systems implemented in healthcare and the fact that nurses constitute the primary providers of patient care [45]. The literature describes various technology acceptance models aimed at understanding technology use, acceptance, or rejection. However, several of these models have failed to provide consistent predictive capabilities for technology acceptance and use in healthcare settings, largely due to the complex interaction of social, organizational, and cultural factors [35,37]. As a result, empirical evidence regarding nurses’ readiness to adopt AI technologies remains fragmented and highly context-dependent, highlighting the need for further applied research that simultaneously examines digital competence, attitudes, and intention to use AI in clinical practice. These perspectives align with broader technology adoption and digital competence research, which emphasizes the role of user readiness, training exposure, and organizational context in shaping the adoption of digital health services.

### 1.3. Research Objectives, Hypotheses, and Contribution

The purpose of the present study was to investigate nurses’ attitudes and perceptions toward AI, as well as to assess their level of digital skills. Given the increasing integration of AI-based systems into clinical practice, understanding nurses’ readiness, acceptance, and competence is essential for the effective and sustainable implementation of such technologies in healthcare settings. By focusing on a national healthcare context with limited existing evidence, the study provides context-specific insights into nurses’ readiness for AI adoption and contributes to the growing body of research on digital health workforce preparedness. Specifically, the study sought to address the following research questions related to AI adoption in nursing practice: (i) What is the level of nurses’ basic digital skills, including information literacy, communication, content creation, problem solving, and security? (ii) What is the level of nurses’ intention to use AI and their expectations regarding the adoption of AI in clinical practice? (iii) What is the level of nurses’ knowledge and education concerning AI?

In addition, the study descriptively explored nurses’ exposure to AI technologies and perceptions related to cultural diversity, as contextual factors influencing familiarity with AI in healthcare environments. From an applied sciences perspective, artificial intelligence in healthcare should be understood not merely as a conceptual innovation, but as a socio-technical system whose successful implementation depends on human–technology interaction, user competence, and organizational readiness. Nurses represent a primary group of end-users of AI-enabled systems, including clinical decision support tools, predictive analytics, automated monitoring systems, and digital documentation platforms. Their digital skills, perceptions, and readiness directly influence whether such technologies are integrated effectively into clinical workflows or remain underutilized. Consequently, empirical evidence on nurses’ readiness for AI adoption constitutes a critical applied prerequisite for the design, implementation, and scaling of AI-based solutions in real-world healthcare settings.

Drawing on prior research on digital competence and technology acceptance in healthcare, the hypotheses below are based on the assumption that the adoption of AI technologies is influenced not only by technical exposure, but also by individual digital skills, educational attainment, and professional background, which collectively shape readiness for AI adoption and perceived usefulness of AI-enabled systems. Accordingly, and in line with relevant research approaches and the existing literature, the present study formulates the following hypotheses:

**H1.**  *Nurses possess basic digital skills relevant to the use of AI-based products and services.*

**H2.**  *Nurses’ attitudes and perceptions toward the adoption of AI differ significantly as a result of demographic characteristics, educational level, and professional experience.*

**H3.**  *Nurses’ knowledge of AI applications is limited due to insufficient education and professional training, while recognizing the necessity of AI-related education.*

The present study contributes to the applied AI and digital health literature in three ways. First, it provides empirical evidence on nurses’ digital skills and AI readiness within a public healthcare context, where large-scale AI deployment is increasingly anticipated but unevenly implemented. Second, it simultaneously examines digital competence, perceptions of professional impact, and readiness for AI adoption, offering a more integrated view of human factors influencing AI implementation. Third, by identifying demographic and professional characteristics associated with lower readiness, the study generates actionable insights for targeted training, workforce planning, and the design of nurse-centered AI adoption strategies.

The remainder of this paper is organized as follows. Section 2 presents the study design, setting, sampling procedure, and data collection instrument, as well as the statistical analyses performed. Section 3 presents the main findings of the study. Section 4 discusses the results in relation to previous literature and highlights implications for nursing practice, education, and policy. Finally, Section 5 concludes the paper and proposes directions for future research.

## 2. Materials and Methods

### 2.1. Study Design

This study was designed as a quantitative, observational, cross-sectional investigation aimed at examining nurses’ attitudes toward artificial intelligence (AI) and assessing their digital skills in public healthcare settings. The cross-sectional design enabled the exploration of associations between nurses’ demographic and professional characteristics and their responses regarding AI at a single point in time, without intervention or manipulation of variables.

The methodological framework was developed to support both descriptive and inferential statistical analyses within an applied nursing workforce research context, providing an applied perspective on nurses’ readiness to adopt AI technologies in clinical practice.

**Figure 1:**
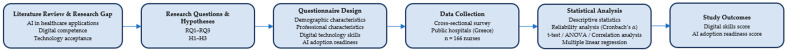
Conceptual research framework and methodological workflow of the study, illustrating the progression from literature review and research questions to questionnaire design, data collection, statistical analyses, and key study outcomes.

As shown in Figure 1, the study followed a structured workflow consisting of the following steps: (1) definition of the research objectives and hypotheses based on the literature review and relevant technology adoption frameworks; (2) development of the questionnaire, including selection of variables and items informed by prior studies and validated instruments; (3) translation and cultural adaptation of questionnaire items into Greek, where applicable; (4) pilot testing and finalization of the instrument; (5) ethical approval and implementation of informed consent procedures; (6) data collection through an anonymous survey; and (7) statistical analysis, including descriptive statistics, group comparisons, correlation analysis, and regression modeling to identify predictors of nurses’ readiness for AI adoption.

### 2.2. Ethical Issues

The study was approved by the Institutional Review Board (IRB) of the postgraduate program in Health Care Management at the Hellenic Open University (approval reference number: 161808/16 October 2024). The research was conducted in accordance with the ethical standards of the Declaration of Helsinki. All participants gave informed consent and were assured that participation was voluntary and could be withdrawn at any time.

### 2.3. Sample and Data Collection

The study sample consisted of 166 nurses employed in two public healthcare institutions in Greece, specifically the University General Hospital of Patras and the General Hospital of Aigio. These hospitals were selected because they are large public healthcare institutions providing a wide range of clinical services and employing nurses across diverse specialties and professional roles.

The inclusion of nurses from two distinct hospital environments aimed to enhance the variability of professional experiences and clinical contexts, thereby strengthening the representativeness of the sample with respect to nursing practice in public healthcare institutions.

Participants were selected through a structured recruitment process across multiple hospital departments, aiming to capture variation in clinical roles, professional experience, and care settings. Although formal probability sampling was not feasible due to organizational constraints, efforts were made to reduce selection bias by inviting nurses from diverse departments and employment positions.

Data collection was conducted over approximately two months, from 6 December 2024 to 14 February 2025. During this period, a total of 170 questionnaires were distributed across the two hospitals. Specifically, 140 questionnaires were collected from the University General Hospital of Patras and 30 questionnaires from the General Hospital of Aigio. Of these, 166 questionnaires were fully completed and included in the final analysis. The overall questionnaire design and structure, including study domains and sources of adopted items, are summarized in Table 1.

### 2.4. Instruments

#### 2.4.1. Study Design and Questionnaire Structure

The present study adopted a quantitative research approach and employed a structured questionnaire as the primary data collection instrument. This approach was considered appropriate for systematically capturing nurses’ perceptions, attitudes, and self-assessed competencies related to artificial intelligence (AI) and digital skills, while enabling statistical analysis and comparisons across participant subgroups. The questionnaire consisted of four sections: (i) demographic characteristics, (ii) professional characteristics, (iii) digital technology skills, and (iv) attitudes toward and readiness for adopting AI in clinical practice.

Questionnaire items were assessed using five-point Likert-type scales, which are widely applied psychometric tools in social and health sciences research for measuring attitudes, perceptions, and levels of agreement with specific statements [48]. Responses ranged from “Strongly disagree” to “Strongly agree” (or, where applicable, from “Not at all” to “Very much”), allowing graded evaluation of participants’ views and self-assessments.

Variables were selected based on the literature review and prior empirical studies examining AI readiness and digital competence among healthcare professionals, as well as established technology acceptance and trust frameworks. Specifically, nurses’ readiness for AI adoption was conceptualized as a behavioral intention construct influenced by perceived usefulness/ease of use, trust, ethical considerations, and organizational support. In addition, digital competence was included as a prerequisite capability enabling effective engagement with AI-enabled tools. Demographic (e.g., age, gender) and professional characteristics (e.g., years of experience, role, workplace setting) were included as potential predictors of readiness based on prior findings indicating subgroup differences in technology adoption.

#### 2.4.2. Reliability and Internal Consistency

The reliability of the questionnaire was evaluated using Cronbach’s alpha coefficient, which is commonly employed to assess internal consistency and the extent to which items within a scale measure the same underlying construct. This procedure ensured that the instrument demonstrated acceptable reliability for the purposes of quantitative analysis.

#### 2.4.3. Questionnaire Content

The questionnaire consisted of the following sections: (i) demographic characteristics, including gender, age, marital status, and educational level; (ii) professional characteristics, including years of professional experience, employment status, area of clinical practice, duration of employment in the hospital, possession of an administrative position, and certification of computer literacy; and (iii) Likert-scale items assessing: (a) self-evaluation of digital skills related to computer use, including information skills, communication skills, software use and content creation, problem-solving skills, and security-related skills; and (b) exposure to AI and cultural diversity, reflecting participants’ interaction with and familiarity with AI-related technologies in healthcare environments.

#### 2.4.4. Validated Scales and Adopted Items

To enhance the methodological rigor and validity of the instrument, selected items were adopted from previously validated studies: (a) 5 items were derived from the study by Karvouniari et al. [39] and were used to examine and assess the digital skills of Greek healthcare professionals, based on validated digital competence indicators, (b) 5 items were adapted from a previously developed instrument assessing intention to use AI in healthcare [40], (c) 7 items were drawn from the studies by Shinners et al. [46,47]. These items correspond to the Shinners Artificial Intelligence Perception (SHAIP) tool and were used to assess healthcare professionals’ perceptions of AI. The structure of the questionnaire and the sources of the adopted items are summarized in Table 1.

The translation and cultural adaptation process followed a forward–backward translation approach, with independent translation into Greek and subsequent review to ensure conceptual equivalence. The adapted questionnaire was piloted in a small sample of nurses to assess clarity and comprehensibility prior to data collection.

For the purposes of analysis, questionnaire items were grouped into composite scales reflecting the main study constructs. Specifically, the SHAIP items were analyzed as three subscales: perceived professional impact of AI, perceived readiness for AI adoption, and expectations/education regarding AI. In addition, items related to exposure to AI and cultural diversity were analyzed as a separate descriptive domain and were not included in inferential or regression analyses. The intention-to-use-AI items were examined within the correlation analysis alongside the main scale scores but were not retained as dependent variables in multivariable regression models. This analytical grouping ensured alignment between the questionnaire structure, scale construction, and statistical analyses. Each composite scale was constructed by grouping items according to their conceptual alignment with the underlying constructs, as defined in the original instruments and adapted for the purposes of this study. No formal psychometric validation (e.g., factor analysis) was conducted in the present study, and the scales were used for exploratory purposes.

### 2.5. Analyses

Categorical variables were summarized using absolute and relative frequencies, whereas continuous variables were described using means and standard deviations (and medians and ranges where appropriate). The Kolmogorov–Smirnov test was applied to assess the normality of the distribution of continuous variables, which supported the use of parametric statistical tests.

Independent variables included nurses’ demographic and professional characteristics, while dependent variables comprised the scores obtained from the study scales. Total years of professional experience and years of experience in the specific hospitals were strongly correlated (r = 0.90, *p* < 0.001); therefore, only total years of professional experience was retained in subsequent analyses to avoid multicollinearity. Due to limited variability (n < 10) within several categories, the variables area of employment and administrative position were excluded from correlation analyses.

Bivariate analyses were selected according to variable type and study design. Independent-samples *t*-tests were used to compare outcome scores between two-group variables, while one-way ANOVA was applied for variables with more than two categories to examine differences in mean outcome scores across independent groups. Pearson correlation coefficients were used for associations between two continuous variables, and Spearman correlation coefficients were used when at least one variable was ordinal.

When bivariate analyses indicated that more than two independent variables were significantly associated with a dependent variable, multivariable linear regression models were fitted to examine predictors of digital skills and readiness for AI adoption. The intention-to-use-AI variable was not included as a dependent variable in multivariable regression models, as it was conceptually examined as an attitudinal outcome within correlation analyses rather than as a primary predictive endpoint. Regression results are presented as coefficients (b), 95% confidence intervals, and *p*-values. Statistical significance was set at *p* = 0.05. All analyses were performed using IBM SPSS Statistics, version 28.0.

For interpretative purposes, scale scores were evaluated descriptively based on the distribution of item responses. The qualitative labels (e.g., ‘low’, ‘moderate’, ‘high’) were used to summarize overall patterns of responses rather than to reflect predefined cut-off thresholds.

## 3. Results

### 3.1. Participant Characteristics

The study sample consisted of 166 nurses. The majority of participants were female (88.6%), older than 35 years (82.9%), and married (57.8%). Regarding educational attainment, 30.7% of the nurses were graduates of secondary education, while 69.3% had completed tertiary education. Among those with tertiary education, 21.7% additionally held a Master’s or Doctoral degree.

With respect to professional experience, nearly half of the nurses (48.7%) reported more than 20 years of total service, whereas 51.3% had 20 years or less of professional experience. The mean total years of service were 17.8 years, indicating a sample with substantial clinical experience. In terms of employment status, 69.9% of participants were permanent employees, 18.1% were contract employees, and 12.0% were engaged in specialization training.

Regarding employment field, 34.3% of nurses were employed in clinical departments, 33.7% in other hospital departments, 18.7% in Intensive Care Units, 9.0% in the laboratory sector, and 4.2% in the Emergency Room. Only a small proportion of participants (6.0%) held a management or administrative position at the time of the study.

Finally, concerning digital qualifications, 39.8% of nurses reported possession of a computer literacy certificate, while 60.2% did not hold such certification. All demographic and professional characteristics of the study population are summarized in Table 2.

### 3.2. Reliability of the Measurement Scales

The internal consistency of all questionnaire scales was assessed using Cronbach’s alpha. The digital skills scale demonstrated very good reliability, while the remaining scales showed acceptable internal consistency, supporting their suitability for subsequent analyses. Cronbach’s alpha, for each of the scales, is presented in Table 3.

Although two scales demonstrated Cronbach’s alpha values slightly below the conventional threshold of 0.70, this indicates moderate internal consistency and suggests that the corresponding results should be interpreted with caution. Given the exploratory nature of the study and the adaptation of instruments to a new context, these values were considered acceptable, while also highlighting the need for further validation in future research.

### 3.3. Descriptive Results: Digital Skills and AI Readiness/Attitudes

Results are presented following the structure of the questionnaire domains to facilitate interpretation from a clinical nursing perspective. Percentages reported correspond to the combined proportion of respondents selecting “agree” or “strongly agree” on the Likert scale. Variables related to exposure to AI and cultural diversity were analyzed descriptively and were not included in inferential or regression analyses. Descriptive results across all study domains are summarized in Table 4.

The descriptive presentation of results follows the analytical grouping of questionnaire domains, whereby composite scales were constructed based on conceptually related items as described in Section 2.4.4.

Overall, findings indicate moderate digital skills and comparatively lower readiness for AI adoption. Exposure to AI technologies in daily life and perceived institutional preparedness were limited, whereas expectations regarding AI’s future impact and its potential to improve efficiency were more pronounced. Notably, the contrast between moderate digital skills and low readiness for AI adoption highlights a critical gap in AI-specific training and preparedness.

### 3.4. Bivariate Associations with Digital Skills and Readiness for AI Adoption

To examine potential differences in key study outcomes across demographic and professional characteristics, bivariate analyses were conducted using independent-samples *t*-tests, one-way ANOVA, and Pearson correlation coefficients. Table 5 summarizes the statistically significant bivariate associations identified. For clarity and brevity, only significant findings are shown.

Digital skills were significantly correlated with age and years of experience (negative associations) and with educational level (positive association). In addition, digital skills differed significantly according to computer literacy certification status, marital status, and employment status.

Readiness for AI adoption was significantly correlated with years of professional experience. Other bivariate comparisons were not statistically significant.

### 3.5. Correlation Analysis

Pearson correlations were computed between the main scale scores and revealed several statistically significant associations. Higher digital skills were associated with greater perceived readiness for AI adoption and greater intention to use AI. Similarly, higher perceived professional impact of AI was associated with both greater readiness and greater intention to use AI. Although the identified associations were statistically significant, their practical implications should be interpreted cautiously, as the relatively low explained variance indicates that additional unmeasured factors likely influence readiness for AI adoption. In addition, higher perceived readiness for AI adoption was associated with greater intention to use AI. To maintain a concise presentation, only statistically significant correlations are reported.

### 3.6. Regression Analysis: Predictors of Readiness for AI Adoption

Multivariable linear regression analyses were conducted following bivariate screening (*p* < 0.20) to identify factors associated with the main study outcomes. All regression models were specified using continuous outcome variables derived from composite scale scores, with independent variables entered simultaneously. Model assumptions were assessed prior to analysis, and no violations were detected. Statistically significant predictors (*p* < 0.05) are summarized below:

- Digital skills were significantly associated with total years of professional experience (b = −0.13, 95% CI: −0.19 to −0.07, *p* < 0.001), educational level (b = 0.57, 95% CI: 0.37 to 0.77, *p* < 0.001), and possession of a computer literacy certificate (b = 0.33, 95% CI: 0.01 to 0.65, *p* = 0.041), collectively explaining 32.5% of the variance in digital skills scores.
- Perceived professional impact of AI was positively associated with educational level (b = 0.57, 95% CI: 0.37 to 0.77, *p* < 0.001), explaining 5.6% of the variance.
- Perceived readiness for AI adoption was negatively associated with total years of professional experience (b = −0.08, 95% CI: −0.12 to −0.02, *p* = 0.006), accounting for 3.9% of the variance in readiness scores.
- Perceived positive impact of AI on healthcare delivery was positively associated with educational level (b = 0.57, 95% CI: 0.37 to 0.77, *p* < 0.001), explaining 4.5% of the variance.

Although intention to use AI was significantly associated with digital skills and perceived readiness in bivariate analyses, it was not retained as a dependent variable in the final multivariable regression models.

## 4. Discussion

Given the cross-sectional design, the analytical approach focused on identifying significant associations and predictors rather than modeling causal mediation pathways. From a nursing practice perspective, the results provide insights into workforce readiness for integrating AI-enabled tools into everyday clinical workflows and should be interpreted within the broader context of nursing informatics and workforce development, where digital competence increasingly shapes clinical decision-making and professional practice. The discussion that follows is structured around the key findings presented in Section 3. Specifically, descriptive findings indicated moderate digital skills but low readiness for AI adoption (Table 4), while bivariate analyses highlighted significant associations between digital skills and demographic/professional characteristics (Table 5). Correlation analysis further demonstrated that higher digital skills were positively associated with perceived readiness for AI adoption and intention to use AI, and regression modeling identified educational level, professional experience, and computer literacy certification as key predictors of digital skills, whereas professional experience emerged as a negative predictor of readiness for AI adoption. These results are interpreted below in relation to previous evidence and their implications for nursing practice, education, and policy.

### 4.1. Digital Skills and Readiness for AI Adoption (Hypothesis 1)

As shown in Table 4 and Table 5, nurses reported moderate digital skills, while readiness for AI adoption remained low. Hypothesis 1 proposed that nurses possess basic digital skills when exposed to AI-based products and services. The findings indicate that nurses indeed demonstrate moderate digital skills, particularly in information-searching, communication, software use, problem solving, and security features, but that foundational knowledge gaps remain evident. Nurses with greater professional experience, higher educational attainment, and a computer literacy certificate exhibited significantly higher digital skills. Importantly, these digital competencies were associated with greater perceived readiness for AI adoption, more favorable perceptions of AI’s professional impact, and greater intention to use AI. These outcomes echo broader trends in the literature, where digital competence is frequently identified as a core enabler of technology adoption among healthcare professionals [20,21,49].

Although previous research highlights the potential of digital skills to support broader technology implementation, it also notes a reliance on support services rather than proactive engagement with digital tools [21]. This underscores the importance of structured digital training and continuous professional development tailored to AI use in clinical environments. From a practical perspective, these findings suggest that improving AI-specific training may have a meaningful impact on nurses’ readiness, even when general digital competence is already present.

### 4.2. Demographic and Professional Differences in AI Perceptions (Hypothesis 2)

Consistent with the bivariate and regression analyses (Table 5; Section 3.6), educational level and professional experience were the main factors associated with AI perceptions and readiness. Hypothesis 2 predicted significant differences in nurses’ attitudes toward AI adoption according to demographic and professional characteristics. The results confirmed that a higher educational level consistently predicted more positive perceptions of AI’s impact, while greater professional experience was associated with lower perceived readiness for AI adoption. These patterns are broadly consistent with previous studies reporting differences in attitudes toward emerging technologies according to educational and professional characteristics [29,50].

The observed differences highlight the need for targeted educational strategies that address not only technical skills, but also confidence and perceived relevance of AI technologies among diverse nursing cohorts. The negative association between longer professional experience and perceived readiness for AI adoption may reflect cumulative exposure to established clinical routines, heightened perceptions of risk associated with technological change, or limited access to structured digital upskilling opportunities over time. Importantly, this finding does not imply resistance among experienced nurses; rather, it underscores the need for tailored training approaches that acknowledge prior professional expertise while fostering confidence in AI-supported clinical practice.

### 4.3. Knowledge, Education, and Training Needs in AI (Hypothesis 3)

The descriptive findings indicate limited AI knowledge and training preparedness (Table 4), supporting the need for structured education. Hypothesis 3 focused on nurses’ knowledge of AI and their recognition of the need for training. The findings partially support this hypothesis: while nearly one-third of nurses reported a basic understanding of AI functions, overall readiness was low, and only a small fraction felt adequately trained to use AI effectively in individualized care planning. This limited AI-specific knowledge and training readiness mirrors findings in broader health professions settings, where knowledge gaps and a lack of formal educational pathways remain significant barriers to effective implementation [30,51,52].

Moreover, although nurses recognize the potential of AI to enhance care delivery, the absence of structured educational programs hampers readiness and may contribute to underutilization of AI tools. Prior reviews emphasize the importance of embedding AI training into health professionals’ education to bridge this gap [25]. Without such preparation, nurses may struggle to leverage the full potential of AI in practice, potentially limiting improvements in efficiency, safety, and personalization of care [53,54].

### 4.4. Limitations

Several limitations of this study should be acknowledged. First, the cross-sectional design precludes causal inference; therefore, longitudinal studies are needed to examine how nurses’ digital skills, perceptions, and readiness for AI evolve over time. Second, the reliance on self-reported measures may introduce reporting or social desirability bias, particularly in the assessment of digital competence, where participants may overestimate or underestimate their actual skills. Third, the study employed non-probability sampling and was conducted in two public hospitals within a specific nursing practice context, which may limit the generalizability and representativeness of the findings with respect to the broader national healthcare system. These sampling characteristics should be taken into account when interpreting the findings, particularly regarding their transferability to other healthcare settings or national contexts.

Although efforts were made to include participants from diverse clinical departments, the sample may not fully capture variability across different institutional settings and regions. In addition, the findings may be influenced by the specific cultural and organizational context of the Greek healthcare system, which may shape attitudes toward technology and AI adoption. Fourth, while the instrument captured basic exposure to AI and self-reported understanding of AI, it did not assess more advanced dimensions of AI literacy, such as familiarity with specific clinical AI applications or ethical and regulatory considerations.

In addition, the study did not include formal factor-analytic validation of the adapted instrument, which constitutes an important limitation and restricts the ability to fully confirm the underlying construct structure. Furthermore, the use of adapted scales without formal psychometric validation in the specific study context may affect the robustness of the derived constructs.

The relatively low proportion of variance explained by some regression models, particularly for readiness for AI adoption, suggests that important influencing factors such as organizational culture, leadership support, digital infrastructure, and institutional readiness, which likely play a central role in shaping nurses’ readiness for AI adoption were not captured in the present study and should be explored in future research. Future studies should further investigate these factors, assess specific AI tools used in Greek clinical settings (including usability, workflow integration, and clinical impact), and conduct multi-center studies with larger and geographically diverse samples to enhance generalizability.

### 4.5. Broader Context and Implications

The findings of this study should be interpreted within the broader trajectory of AI integration into healthcare systems globally. AI technologies are rapidly reshaping clinical workflows, operational processes, and healthcare delivery models, with evidence suggesting transformational potential in areas such as predictive analytics, decision support, and personalized medicine [31,33,55]. For nursing practice specifically, AI holds promise to enhance documentation, patient monitoring, and clinical decision-making through advanced data analysis and real-time support systems [6,39].

Yet, realizing this potential requires attention to structural, educational, and ethical dimensions of adoption. Systematic reviews of AI adoption in healthcare report persistent barriers including infrastructure limitations, limited training, workflow misalignment, and concerns about transparency and accountability [1,31]. These challenges are particularly salient in the nursing context, where front-line professionals must balance clinical demands with adoption of complex technologies. Addressing these challenges requires not only individual-level training but also institutional commitment, including the development of structured implementation strategies, leadership engagement, and the integration of AI competencies into organizational policies and professional development frameworks.

Furthermore, broader healthcare research has underscored the importance of responsible and equitable AI deployment. Ethical deployment guidelines, such as the FUTURE-AI framework, recommend prioritizing fairness, explainability, usability, and robustness to ensure that AI systems are accepted by clinicians, trusted by patients, and supportive of clinical decision-making, without exacerbating existing disparities or undermining professional skills [56]. A growing body of evidence further suggests that, beyond technical performance, successful AI adoption depends critically on trust, interpretability, and clinician engagement [43]. Accordingly, policies aimed at expanding access to AI education, fostering interdisciplinary collaboration, and aligning AI tool development with nursing workflows are essential. In this regard, international frameworks emphasize that effective AI adoption in healthcare requires coordinated organizational strategies, interdisciplinary collaboration, transparency, and sustained human oversight to ensure safety, accountability, and ethical deployment in clinical practice [32].

These broad considerations suggest that while nurses may express moderate readiness and positive attitudes toward AI, systematic investments in education, organizational alignment, and ethical governance are critical to ensure that AI enhances care delivery without undermining core nursing functions or professional autonomy.

## 5. Conclusions

The present study found that nurses demonstrated moderate digital skills, with 59.1% reporting competence in email/video communication but only 27.2% indicating adequate use of digital security tools. Exposure to AI was limited (18.0% reported using AI products/services in daily life), and readiness for AI adoption in clinical practice was comparatively lower, as only 7.8% of participants considered health professionals adequately prepared for AI and 7.2% reported adequate AI training. In bivariate and multivariable analyses, digital skills were significantly associated with educational level and computer literacy certification, while longer professional experience was consistently associated with lower perceived readiness for AI adoption (b = −0.08, *p* = 0.006). Overall, these results suggest a gap between general digital competence and preparedness for AI-enabled clinical implementation, supporting the need for targeted AI education and workforce development strategies. These findings should be interpreted within the context of the study sample and design. Future nursing research should further explore how digital competence development and AI literacy interventions influence clinical adoption and professional practice outcomes.

These findings suggest that the effective and sustainable adoption of AI in healthcare requires not only technological availability but also systematic investment in workforce preparation. Successful AI integration should be supported by: (a) organizational strategies that promote supportive work cultures, updated procedures, and training programs for clinical guidance; (b) the development of interdisciplinary collaboration models that actively involve frontline healthcare professionals alongside technical experts; and (c) mechanisms that ensure coordination, transparency, and human oversight, while promoting safety, accountability, and trust in AI-supported decision-making. From an organizational perspective, these findings highlight the need for institutional strategies that support structured training, leadership engagement, and the integration of AI competencies into professional development frameworks for nurses.

From an applied perspective, AI implementation strategies in healthcare should be accompanied by role-specific training programs for nurses, integration of AI literacy into continuing professional education, and organizational policies that support gradual and transparent adoption of AI tools. Aligning AI system design with nursing workflows and competencies may enhance acceptance, reduce uncertainty, and promote the safe and effective use of AI in everyday clinical practice.

## Figures and Tables

**Table 1:** Questionnaire design and structure.

Questionnaire Domain	Content/Variables	No. of Items	Source/Reference
Demographic characteristics	Gender, age, marital status, educational level	4	Study-designed
Professional characteristics	Years of experience, employment status, clinical area, years in current hospital, management position, computer literacy certificate	6	Study-designed
Digital skills	Information literacy, communication, software/content creation, problem solving, security	5	Karvouniari et al. [39]
Intention to use AI	Willingness/intention to use AI applications in clinical practice	5	Armeni [40]
AI perceptions (SHAIP tool)	Perceived professional impact, perceived readiness, expectations/education	7	Shinners et al. [46,47]

**Table 2:** Demographic and professional characteristics of the study sample (n = 166).

Characteristic	n (%)
Gender	
Male	19 (11.4)
Female	147 (88.6)
Age (years)	
18–24	15 (9.0)
25–34	30 (18.1)
35–44	32 (19.3)
45–54	47 (28.3)
55–64	42 (25.3)
Family Status	
Unmarried	54 (32.5)
Married	96 (57.8)
Separated	16 (9.6)
Educational level	
Secondary education	51 (30.7)
Higher education	79 (47.6)
Master’s/Doctoral degree	36 (21.7)
Total years of service	
1–5	49 (29.5)
6–10	15 (9.0)
11–15	8 (4.8)
16–20	13 (7.8)
21–25	19 (11.4)
26–30	20 (12.0)
>30	42 (25.3)
Employment contract	
Permanent staff	116 (69.9)
Contract employee	30 (18.1)
Specialization	20 (12)
Employment field	
ICU	31 (18.7)
Clinical departments	57 (34.3)
Laboratory sector	15 (9.0)
Emergency Room (ER)	7 (4.2)
Other departments	56 (33.7)
Years of service	17.8 ± 13.4
Management position	
No	156 (94.0)
Yes	10 (6.0)
Computer literacy certificate	
Yes	66 (39.8)
No	100 (60.2)

Note: Data are presented as number (percentage), except for years of service, which are presented as mean ± standard deviation.

**Table 3:** Internal consistency of the questionnaire scales.

Scale	Cronbach’s α
Digital skills (computer-related)	0.873
Exposure to AI and cultural diversity	0.710
Perceived professional impact of AI	0.726
Perceived readiness for AI adoption	0.662
Expectations and education regarding AI	0.656

**Table 4:** Nurses’ responses across study domains (% agree/strongly agree; n = 166).

Domain	Item	n (%)
Digital skills and computer use	Use of computers for email, video calls, and social networking	59.1
	Use of copy–paste tools in documents	51.2
	Searching health information from public authority websites	48.8
	Creating presentations/attending online courses	29.6
	Use of security software (antivirus, firewall, etc.)	27.2
Exposure to AI and cultural diversity	Use of AI products/services in daily life	18.0
	Basic understanding of AI and its functioning	28.9
	Prior troubleshooting of AI products/services	11.8
	Cultural background influences attitudes toward AI	27.7
	Cultural diversity provides unique perspectives on AI	38.6
Perceived professional impact of AI	AI will change the nursing role in the future	50.0
	AI improves patient care delivery	42.2
	AI improves clinical decision-making	37.9
	AI reduces healthcare costs	36.7
	AI will assume part of the nursing role	21.7
Perceived readiness for AI adoption	Health professionals are adequately prepared for AI	7.8
	Adequate AI training for individualized care planning	7.2
Expectations and education regarding AI	AI will have a substantial future impact on specialization	46.4
	AI can solve complex problems and save time/cost	54.9
	AI improves decision-making processes and efficiency	39.7
	AI will shift or create new job opportunities	37.3
	Significant challenges in AI adoption	28.3
	Nursing institutions are adequately prepared	7.2

**Table 5:** Summary of significant bivariate analyses for digital skills and AI adoption readiness.

Outcome	Independent Variable	Test	Result	*p*-Value
Digital skills	Age group	Spearman’s rho	ρ = −0.322	<0.001
	Educational level	Spearman’s rho	ρ = 0.441	<0.001
	Years experience	Pearson’s r	r = −0.377	<0.001
	Computer literacy (yes)	*t*-test		<0.001
	Marital status (unmarried/separated)	*t*-test		<0.01
	Employment (in specialization training)	ANOVA		<0.001
AI adoption readiness	Years experience	Pearson’s r	r = −0.202	<0.05

## Data Availability

The raw data supporting the conclusions of this article will be made available by the authors on request due to ethical restrictions related to participant confidentiality and institutional approval conditions.

## Public Involvement Statement

No public involvement in any aspect of this research.

## Guidelines and Standards Statement

This manuscript was drafted in accordance with the STROBE (Strengthening the Reporting of Observational Studies in Epidemiology) guidelines for cross-sectional studies.
